# A Route to the Colorimetric Detection of Alpha-Fetoprotein Based on a Smartphone

**DOI:** 10.3390/mi15091116

**Published:** 2024-08-31

**Authors:** Junjie Liu, Qingfubo Geng, Zhaoxin Geng

**Affiliations:** 1 School of Information Engineering, Minzu University of China, Beijing 100081, China; 17301253@muc.edu.cn (J.L.); 23012784@muc.edu.cn (Q.G.); 2 Key Laboratory of Ethnic Language Intelligent Analysis and Security Governance of MOE, Minzu University of China, Beijing 100081, China

**Keywords:** biosensor, colorimetric detection, microfluidics

## Abstract

Alpha-fetoprotein (AFP) is a key marker for early cancer detection and assessment. However, the current detection methods struggle to balance accuracy with the need for decentralized medical treatment. To address this issue, a new AFP analysis platform utilizing digital image colorimetry has been developed. Functionalized gold nanoparticles act as colorimetric agents, changing from purple-red to light gray-blue when exposed to different AFP concentrations. A smartphone app captures these color changes and calculates the AFP concentration in the sample. To improve detection accuracy, a hardware device ensures uniform illumination. Testing has confirmed that this system can quantitatively analyze AFP using colorimetry. The limit of detection reached 0.083 ng/mL, and the average accuracy reached 90.81%. This innovative method enhances AFP testing by offering portability, precision, and low cost, making it particularly suitable for resource-limited areas.

## 1. Introduction

Alpha-fetoprotein (AFP) is a glycoprotein that is primarily synthesized by the fetal liver and yolk sac and is found in relatively low levels in other organs [1]. It is important to note that AFP levels in a healthy person usually stabilize after the age of two. Nonetheless, when liver cells become cancerous, the AFP concentration increases significantly [2]. AFP is a commonly used biomarker for the detection of hepatocellular carcinoma and testicular and ovarian tumors. Its clinical detection limits vary depending on the detection method and equipment. In general, the normal reference range and clinical detection limits of AFP are as follows: (1) Normal reference range: Normal serum AFP levels in adults are usually below 10 ng/mL. (2) Clinical significance: (A) Mild elevation (10–200 ng/mL): may be associated with chronic liver disease (such as hepatitis, cirrhosis), pregnancy, or other benign diseases. (B) Significantly elevated (>200 ng/mL): highly suspected primary liver cancer or germ cell tumor. In particular, when AFP levels exceed 500 ng/mL, hepatocellular carcinoma can almost be diagnosed [3,4,5,6]. Therefore, AFP levels have become an important marker for early liver cancer screening, assessing treatment effectiveness, and identifying recurrence [7].

Nevertheless, common methods for detecting tumor markers include enzyme-linked immunosorbent assay (ELISA), immunohistochemistry (IHC), radioimmunoassay (RIA), chemiluminescent immunoassay (CLIA), electrochemiluminescence immunoassay (ECLIA), and molecular biology methods (such as polymerase chain reaction (PCR) and next-generation sequencing (NGS)). Each method has its advantages and disadvantages, and the choice of method typically depends on the nature of the marker, the detection requirements, and the equipment and technical expertise available in the laboratory. Traditional detection methods rely on expensive, bulky, benchtop analyzers operated by professionals, and patients often face several days of waiting for test results. Consequently, traditional medical detection technology struggles to meet the needs of early cancer diagnosis and prevention [8].

The development of point-of-care testing (POCT) technology based on smartphones offers a promising solution to these challenges [9,10,11]. POCT emerged in the 20th century, with early examples including test strips for detecting blood glucose and urine glucose [12,13,14]. Over time, the focus of POCT has become more defined, now representing the concept of decentralized medicine. It is characterized by short testing times, low costs, and simple operation. The advent of POCT has significantly alleviated the burden on hospitals and laboratories. Additionally, POCT devices typically provide test results within a few hours, reducing patient waiting times. Consequently, POCT serves as an effective alternative to traditional lab-based testing, particularly in developing countries [15,16].

With the development of information technology and biotechnology, the practical application of POCT involves collecting samples directly from patients in various settings such as hospitals, clinics, community health centers, pharmacies, urgent care centers, and even patients’ homes. The advantages of the wide application of POCT are mainly reflected in the following aspects: (1) Enhanced medical efficiency; (2) Cost reduction; (3) Increased accessibility; (4) Empowered patient self-management; (5) Immediate clinical decisions; and (6) Support for public health initiatives.

Currently, there are four main methods used to detect POCT: brightfield, colorimetry, fluorescence, and electrochemistry [17,18,19,20,21]. Brightfield detection is commonly used to observe cell samples [22]. Fluorescence and electrochemical methods offer the advantage of high accuracy and sensitivity. However, fluorescent reagents require careful storage and experimental conditions, while the preparation and operation of electrochemical devices are complex [20]. Colorimetric sensing is a method that detects the concentration or presence of substances by observing color changes. It is widely used in biology, chemistry, medicine, and environmental fields. Colorimetric sensing based on smartphone platforms utilizes the camera, screen, processor, and other functions of smartphones for acquiring, analyzing, displaying, and transmitting colorimetric sensing data. The advantages of using smartphone-based colorimetric sensing include the following: (1) Portability: It can be conducted anytime and anywhere without the need for professional instruments and equipment, making it suitable for use in homes, hospitals, rural areas, remote locations, and other settings. (2) Real-time monitoring: Smartphones can collect and process colorimetric data in real-time, displaying the results on the screen or transmitting them wirelessly to achieve quick decision-making. (3) Accuracy: The high-resolution cameras and high-performance processors of smartphones help accurately identify and analyze contrast color sensing images, minimizing human error and interference, and enhancing measurement accuracy and reliability. (4) Versatility: Smartphones can combine different colorimetric agents and software to achieve sensing of various substances for different detection needs and applications. (5) Cost-effectiveness: They are more affordable and easier to obtain compared to professional instruments and equipment. Colorimetric agents and software can also be readily prepared and downloaded to achieve low-cost colorimetric sensing.

For the reasons mentioned above, many researchers have proposed AFP detection methods based on colorimetric methods. C. Nietzold and F. Lisdat proposed a scheme for detecting AFP based on the colorimetric principle [23]. In this method, the AFP antibody was coupled to gold nanoparticles. After the corresponding antigen was added, the wine-red colloidal gold would gradually turn gray-blue. Since then, Ying Sun et al. have further improved this method [24]. The research team further improved the detection performance by using lens culinaris agglutinin. In 2017, Qian Xiao proposed a new method [25]. The gold nanoparticles (AuNPs) were employed to synthesize an ultrasensitive probe by dual-film assembly with anti-AFP on the AuNP surface. The introduction of dual-film-modified AuNPs to an enzyme-linked immunosorbent assay (ELISA) resulted in an ultrasensitive signal amplification to quantitatively analyze AFP. Eda A. et al. immobilized functionalized AuNP on nitrocellulose membrane [26]. Then, Image J (V 1.8) was used to further analyze the color change after adding AFP. This method solved the practical problem of AFP detection in POCT, which illustrated that paper-based sensors had good portability. However, one drawback was that the sensor had a shelf life of only four days at room temperature. Therefore, Xuewen Lu’s group presented a new method to reduce the detection limit of AFP using a spherical core-shell gold–silica nanoparticle (AuNP@SiO_2_ NP) structure [27]. The nanoparticles could detect AFP as low as 300 pg/mL, which was 30-fold more sensitive than traditional detection methods. Moreover, this method could detect other cancer markers.

Despite these advancements, a critical issue remains unaddressed: high-precision AFP detection methods still require further processing in the laboratory. Methods operated outside the laboratory can only provide a binary “Yes” or “No” answer. To tackle this, we proposed a portable AFP colorimetric detection system based on a smartphone, combining POCT with AFP detection. The system comprises both hardware and customized software. The hardware platform shields external light interference and provides uniform illumination. The software, developed using Android Studio, calculates the AFP concentration and saves the data. The entire detection process can be completed in 10 min.

## 2. Materials and Methods

### 2.1. Materials and Instruments

All chemicals were analytical-grade reagents and used as received without further purification. Citrate-stabilized AuNP (diameter = 60 nm) was purchased from Nanjing Nanoeast Biotech Co., Ltd. (Nanjing, China). Sodium phosphate buffer (SPB) was purchased from Shanghai Yuanye Biotech Co., Ltd. (Shanghai, China). Phosphate-buffered saline (PBS) was purchased from Suolaibo Biotech Co., Ltd. (Beijing, China). Mercaptopropionic acid (MPA) was purchased from Beijing Bailingwei Technology Co., Ltd. (Beijing, China). N-Hydroxy succinimide (NHS) and N-(3-dimethylaminopropyl)-N-ethylcarbodiimide hydrochloride (EDC) were purchased from Sigma-Aldrich (Shanghai, China). Anti-alpha feta-protein (Anti-AFP) was purchased from Abcam (Cambridge, UK). Alpha-fetoprotein antigen (AFP-antigen) was purchased from Creative BioMart (New York, NY, USA). A mini-ultracentrifuge, mixer, and shaker were purchased from Dragon Laboratory Instruments Limited Company (Beijing, China). Other materials were obtained locally at a market. The spectrometer was purchased from Ocean Optics Company (Largo, FL, USA,). 

### 2.2. Conjugation of Colloidal Gold with Antibodies

The process is as follows:

(1) Firstly, the pipette gun was used to absorb citrate-stabilized AuNPs (diameter = 60 nm, 1 mg/mL) of 200 μL into the centrifuge tube, and then the centrifuge tube was centrifuged with a high-speed centrifuge (parameter: 9300 g, 10 min). Secondly, 150 μL supernatant was removed with a pipette; after this step, 150 μL MPA was added into the centrifuge tube, and then a carboxyl mercaptan layer was introduced with a high-speed centrifuge. Lastly, the sample was left to stand for more than 1 h at 23 °C.
(2) The excess MPA was removed with a high-speed centrifuge, 150 μL of the supernatant was removed after each centrifugation, and the same volume of SPB was put into the centrifuge tube. This step was repeated 2–3 times.
(3) After the last centrifugation, 50 μL EDC and 100 μL NHS were added to the original volume of 200 μL, mixed in, and left for about 30 min.
(4) The excess EDC and NHS were removed with a pipette after centrifugation. This step was repeated 2–3 times. Then, 150 µL of supernatant was absorbed out, and SPB of the same volume was added each time. Then, 2 µL of configured AFP antibody was added after centrifugation, and the centrifuge tube with the sample was placed in a shaking table at 23 °C for 1.5 h.
(5) The excess antibodies were removed after centrifugation twice. Each time, 150 μL of supernatant was taken out. The first time, 150 μL of SPB was added. The second time, 50 μL of SPB was added. The last time, only 50 μL of SPB was added to improve the colloidal gold concentration, since colloidal gold will have a trace loss each time; otherwise, the experimental phenomenon is not obvious.
(6) Finally, the functionalized AuNPs were resuspended in SPB after centrifugation and stored at 4 °C until use.

### 2.3. AuNPs Surface Immune Reaction

The morphology of gold nanoparticles significantly influences their localized surface plasmon resonance (LSPR) scattering spectrum, which in turn affects the color changes observed in colorimetric reactions. Due to this property, many researchers have studied the impact of gold nanostructures on sensing characteristics and the synthesis methods of gold nanoparticles. Michael R. and colleagues have already provided a comprehensive review of this research area [28]. Therefore, in this paper, we will focus on the practical aspects, and we purchased commercially available gold nanoparticles instead of delving into their synthesis methods (as described in Section 2.1 and Section 3.1).

The colloidal-gold surface immune reaction was produced by mixing the functionalized AuNPs and the AFP antigen in a volume ratio of 9:1. The mixture was then incubated for 10 min at approximately 23 °C. 

Throughout the experiment, it was crucial to maintain a stable temperature of about 23 °C. Lower temperatures could result in insufficient antibody incubation, thereby affecting the experiment’s outcomes. Additionally, maintaining appropriate reagent concentrations was vital. Excessively high concentrations of reagents could lead to the direct accumulation of gold nanoparticles.

The stability of the biochemical sensing experiment is a crucial factor. However, it is important to note that the colorimetric reaction based on gold nanostructures has a limited timeframe. All chemical reagents should be prepared within 2 weeks to ensure the experiment can be replicated. The surface activation time of the gold nanostructure should not exceed 1 h, and the bonding time of the antibody with the functionalized nano should generally be less than 4 h and preferably carried out at 4 °C. The combination of the antibody and antigen should be completed within 40 min, and the corresponding colorimetric test should also be performed within a reasonable timeframe. The stability of the chemical reaction time is crucial for the success of the experiment.

## 3. Result and Discussion

### 3.1. AuNPs as Colorimetric Agent

In this experiment, the AuNPs carry a negative charge and remain dispersed in the liquid environment, making the Zeta potential crucial in this process. This directly affects the dispersion, stability, and sensitivity of the nanoparticles, thereby influencing the performance of the sensor [29,30,31]. The impact of the Zeta potential on colorimetric sensing can be summarized as follows:

(i) Particle Dispersion and Stability:

A. Preventing Particle Aggregation: A high Zeta potential (negative value) helps gold nanoparticles remain dispersed in the solution, preventing particle aggregation due to insufficient electrostatic repulsion. A stable dispersion of nanoparticles is vital for the sensitivity of the colorimetric sensor, as aggregation can alter the optical properties of the nanoparticles, especially their localized surface plasmon resonance (LSPR) absorption peak.

B. Controlling and Utilizing Aggregation: In some colorimetric sensing assays, controlling the Zeta potential to induce particle aggregation and generate a color change is central to the detection mechanism. The interaction between target molecules (such as AFP) and the surface of gold nanoparticles can cause changes in the Zeta potential, leading to nanoparticle aggregation and subsequent color changes. This color change can be used for the quantitative detection of the target molecule’s concentration.

In the experiment, all AuNPs have an excess of negative charges on their surfaces, allowing them to adsorb positively charged groups from macromolecules like AFP without compromising the biological properties of the protein. AuNPs not only possess quantum size and surface effects but also exhibit good biocompatibility and low toxicity. The maximum absorption wavelength of gold nanoparticles (with a diameter range of 20–60 nm) lies between 520 and 535 nm. Studies have shown that larger AuNPs exhibit more noticeable color changes when interacting with the corresponding proteins. Therefore, 60 nm AuNPs were chosen for further modification.

(ii) Sensitivity and Specificity:

A. Enhancing Detection Sensitivity: Changes in the Zeta potential can significantly affect the optical properties of nanoparticles, such as the sensitivity of color changes. By optimizing the Zeta potential, the response of the colorimetric sensor to the target analyte can be enhanced. In this experiment, to ensure the repeatability of the results, the charge on the gold nanoparticles used each time must remain consistent, and they must carry enough negative charge to maintain their dispersion in the liquid environment.

B. Improving Specificity: Specific surface functionalization can adjust the Zeta potential to selectively detect target molecules. This helps reduce nonspecific adsorption and background noise, thereby improving the specificity of the sensor. In this experiment, the focus is on effectively detecting AFP, which maintains a consistent positive charge, significantly improving the reliability of the test results.

(iii) Signal Amplification and Visual Detection:

Amplification of Color Change: Nanoparticle aggregation is usually accompanied by a noticeable color change, which forms the basis of visual detection in colorimetric sensors. The change in the Zeta potential induces nanoparticle aggregation, thereby amplifying the color signal and enabling the detection of low-concentration targets. 

Through these mechanisms, the Zeta potential plays a crucial role in the colorimetric sensing of AuNPs, ensuring the sensitivity, specificity, and visual detection effectiveness of the sensor. However, the AuNPs used in this experiment were provided by the merchants, and the results were obtained through repeated experiments. Therefore, AuNPs with a suitable Zeta potential (about −33.2 mV) were selected in the experiment to ensure the effective combination of them with AFP.

**Figure 1:**
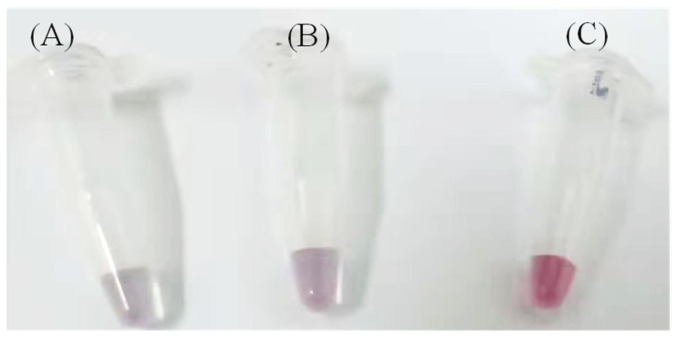
Visual color change of functionalized AuNPs upon addition of AFP at different concentrations. (**A**) 200 ng/mL; (**B**) 100 ng/mL; (**C**) 0 ng/mL.

**Figure 2:**
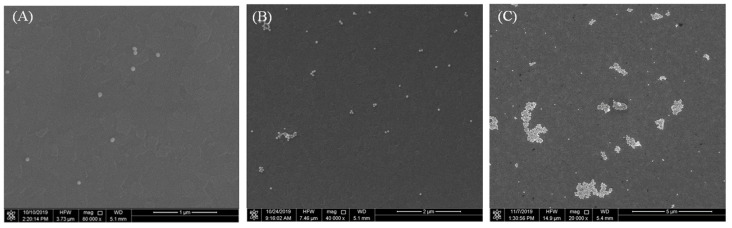
The scanning electron microscope (SEM) of functionalized AuNPs (diameter = 60 nm) on the silicon wafer; (**A**) The AuNPs are in a dispersed state without any modification; (**B**) AuNPs are still in a dispersed state (only a small amount of aggregation) after coupling of colloidal gold with antibodies steps; (**C**) AuNPs accumulated extensively after colloidal-gold surface immune reaction.

**Figure 3:**
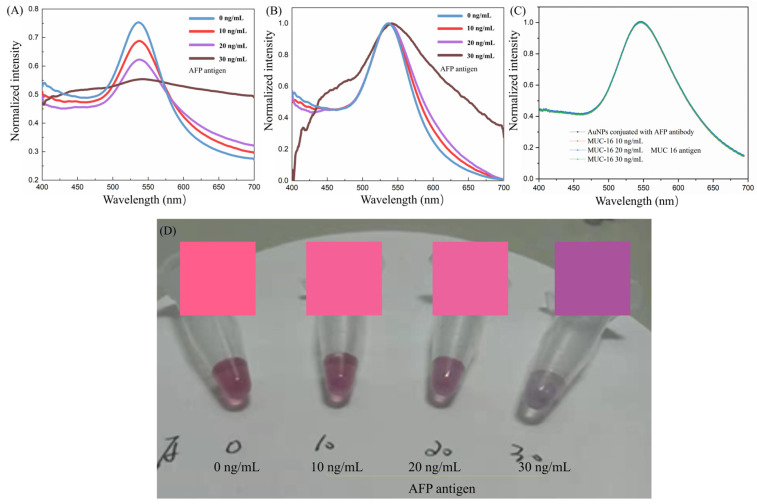
UV/Vis spectra of AuNPs solutions after adding different concentrations of AFP MUC16 and images of AFP experiments, the numbers 0, 10, 20, and 30 represent the addition of AFP antigens of 0, 10, 20, and 30 ng/mL, respectively; (**A**) Initial spectrum of testing AFP; (**B**) Normalized spectrum of testing AFP; (**C**) Normalized spectrum of testing MUC16; (**D**) Actual sample image with different AFP antigen concentrations of 0, 10, 20, and 30 ng/mL and color calculated by Matlab (20 16) program.

Following modification through the aforementioned steps, functionalized AuNPs were combined with AFP, resulting in the gradual transition of the purple-red colloidal gold to a light gray-blue hue, as depicted in Figure 1. This transformation occurs as discrete AuNPs progressively aggregate under the influence of AFP, as illustrated in Figure 2. Both the size and shape of nanoparticles influence the absorption peak. Electric dipole-dipole interactions between neighboring nanoparticles cause color shifts as the interparticle distance decreases (due to analyte conjugation or antibody binding) to less than the average particle diameter, leading to a blue shift. With increasing AFP concentration, the absorbance of the entire system diminishes due to gold nanoparticle aggregation, as demonstrated in Figure 3A.

To further validate the effect of AFP concentration on the sample’s color, the spectral map was converted into the corresponding color image using Matlab. As depicted in Figure 3, the spectrum in Figure 3A was normalized to obtain the image displayed in Figure 3B, which was then imported into a custom Matlab program to generate a color image. The results, along with the original sample image, are presented in Figure 3D. Samples labeled 0, 10, and 20 correspond well with the calculated color; however, sample 30 exhibits a larger error. This discrepancy can be attributed to two factors. Firstly, there may be a discrepancy between the actual sample color and the image captured under external lighting conditions. Secondly, sample 30, having a higher antigen concentration, yields lower peaks, making it challenging for the program to calculate the color accurately. In smartphone-based colorimetric sensing, environmental factors significantly impact measurement results. These factors include lighting conditions, background color, and the surface condition of the object. To improve measurement accuracy, it is generally necessary to control these environmental factors during measurement or use algorithms to compensate for these interferences. Therefore, a small hardware system for placing the smartphone was developed.

To demonstrate the specific expression of the surface functionalization of colloidal gold and the binding of AFP antigen, we used the non-specific binding experiments of MUC-16 (purchased from Sigma-Aldrich (Shanghai, China)) with AFP antibodies and compared them with the control group experiments. The concentration of MUC-16 is the same as the concentration of the AFP antigen. MUC-16, also known as Mucin 16 or CA-125, is a large membrane-bound glycoprotein that is part of the mucin family. Mucins are known for their role in protecting epithelial cells and facilitating various cellular functions. MUC-16 is particularly significant in the context of cancer biology, especially in ovarian cancer. It is also used in the early diagnosis and monitoring of cancer.

Binding of functionalized colloidal gold with AFP antigen: The result is that the functionalized colloidal gold nanoparticles should be highly specific to the AFP antigen and show significant signal changes. When AFP antigens of varying concentrations bind with AFP antibodies, a redshift in the spectral resonance peak is observed. The higher the concentration of AFP antibodies, the more pronounced the redshift (greater ∆λ), indicating effective binding between the AFP antibodies and antigens (as shown in Figure 3A,B).

Binding of functionalized colloidal gold with MUC-16: As a result, functionalized colloidal gold nanoparticles should exhibit very low or nonspecific binding to MUC-16 proteins, with weak or no significant changes in signaling (as shown in Figure 3C).

Control group: The results showed that there should be no specific binding between the unfunctionalized gold nanoparticles and AFP and MUC-16 proteins, and the signal changes should be weak.

### 3.2. Improving the Sensitivity and Portability of Digital Image-Based Colorimetry

The most significant interference factor for colorimetric detection is the influence of external light. Variations in external lighting conditions during image collection can introduce substantial interference, resulting in larger errors in the analysis results. To mitigate this issue, a portable hardware platform was designed. This platform serves to securely hold experimental samples, block out external light, and provide a consistent light source, ensuring more accurate and reliable imaging results.

**Figure 4:**
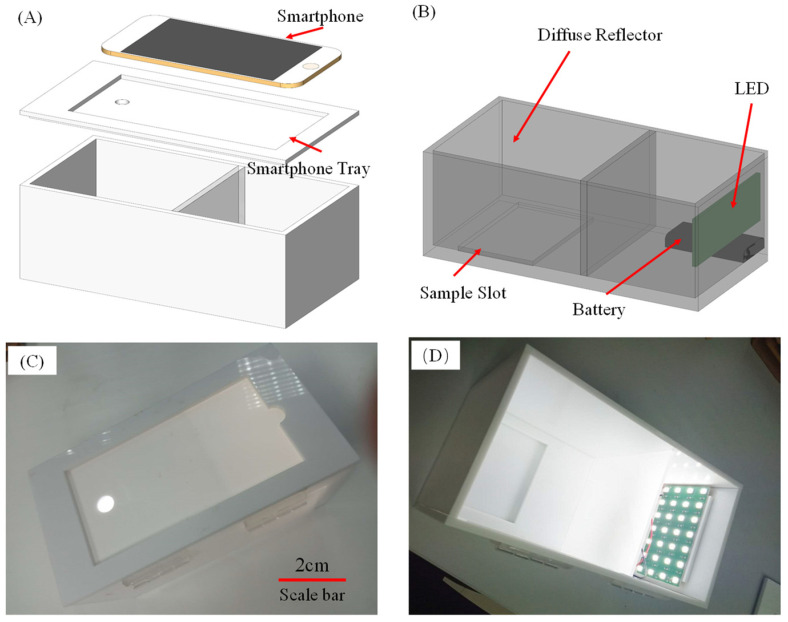
Overall structure diagram of the hardware platform. (**A**,**B**): Design drawing of the hardware platform; the overall structure is cubic, with LED, diffuse reflector sample slot, etc.; (**C**,**D**): Optical image of the hardware platform.

As depicted in Figure 4, the hardware platform, designed using SolidWorks, measures 210 mm in length, 110 mm in width, and 110 mm in height. Constructed from 5 mm thick white polymethyl methacrylate materials, the platform is divided into several parts for ease of processing and then assembled using acrylic glue. Inside the platform, there is a sample placement slot where samples are positioned during experiments. A white LED is situated behind the interior of the platform. The light passes through a diffuse reflection plate in the middle, transforming non-parallel light into uniform illumination, thereby preventing issues such as shadows and uneven lighting caused by point light sources. The entire light source system is powered by 2 AAA batteries, with a switch located on the side of the hardware platform. A smartphone is positioned above the hardware platform, capturing the internal experimental setup through a circular aperture with a diameter of about 1 cm. The left side of the hardware is designed with an “open the door from above” style, facilitating the placement of experimental samples and battery replacement.

The entire hardware platform not only effectively mitigates external light interference but also boasts a low cost. The total cost of the hardware equipment can be kept within USD 5. Additionally, the hardware is convenient, compact, and easy to carry. Importantly, the required components do not necessitate precise devices, making it more conducive to dissemination in areas with limited resources.

In addition to the hardware platform, a custom app developed using Android Studio was utilized. This app calculates the concentration in the sample by analyzing the color change after the addition of AFP. The functionalized AuNPs transitioned from magenta to light gray-blue, resulting in a decrease in color depth. Consequently, the gray space was utilized to establish the relationship between color and sample concentration.

**Figure 5:**
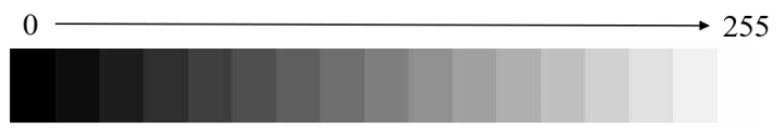
The schematic of grayscale.

Grayscale utilizes black as the reference color, with the object color represented by varying degrees of black saturation, as illustrated in Figure 5. Grayscale values are typically expressed as percentages, ranging from 0% (black) to 100% (white). Each pixel in a grayscale image is stored with an 8-bit non-linear scale, allowing for the representation of 256 grayscale values. This method of saving enables accurate representation without band distortion. Moreover, grayscale images contain only brightness information, eliminating irrelevant color data present in the original image. Consequently, image processing computations are significantly reduced, resulting in faster processing times.

While a smartphone can directly acquire the RGB values of an image, grayscale values need to be derived using the following Formula (1):

Gray value = 0.299 × R + 0.587 × G + 0.114 × B(1)

The functional relationship between the gray value and sample concentration to be measured was fitted by the least-square method. The least-square method finds the best matching function by the least-square value of error. The mathematical definition of the least-square method is $y_{i}=ax_{i}+b+e_{i}$, i∈[1,n]. $e_{i}$ acts as the sample, ($x_{i},\;y_{i}$) is the real value of $\;y_{i}=ax_{i}+b+e_{i}$, and $y_{i}^{\prime }=ax_{i}+b$ is an error value. The square loss function *Q* is

(2) $$Q=e_{i}^{2}=\sum_{i=1}^{n}{\left(y_{i}-y_{i}^{\prime }\right)}^{2}=\sum_{i=1}^{n}{\left(y_{i}-ax_{i}-b\right)}^{2}$$

Take variables a and *b* as the independent variables to find the minimum value of *Q*—that is, take the derivative of a and *b,* respectively, and set them to 0.

(3) $$\frac{\partial Q}{\partial a}=2(a\sum_{i=1}^{n}x_{i}^{2}-\sum_{i=1}^{n}(y_{i}-b)x_{i})=0$$

(4) $$\frac{\partial Q}{\partial b}=2(nb-\sum_{i=1}^{n}(y_{i}-ax_{i}))=0$$

Finally, the value of a and *b* can be found by solving the equations:

(5) $$a=\frac{\sum_{i=1}^{n}y_{i}(x_{i}-\frac{1}{n}\sum_{i=1}^{n}x_{i})}{\sum_{i=1}^{n}x_{i}^{2}-\frac{1}{n}(\sum_{i=1}^{n}x_{i}{)}^{2}}$$

(6) $$b=\frac{1}{n}\sum_{i=1}^{n}\left(y_{i}-ax_{i}\right)$$

**Figure 6:**
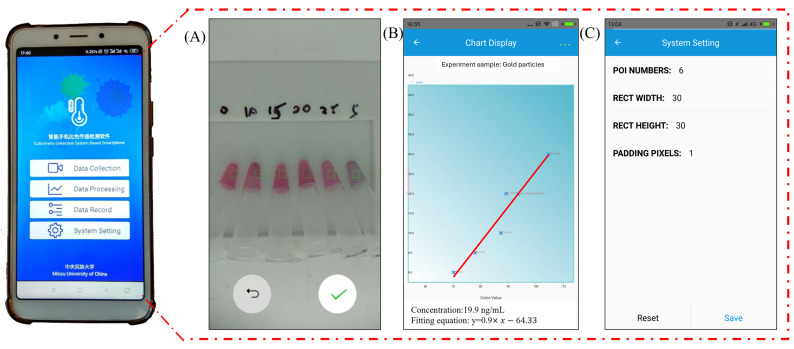
The custom smartphone app 1.1. (**A**) Image capture interface—the green rectangular frame is the framing frame, and the custom app analyzes and calculates the color value in the framing frame; (**B**) The image display interface, which includes the fitted concentration–color equation, equation formula, and concentration value of the sample to be measured; (**C**) Set framing parameters, including number, size, and other information.

In the experimental process, the user inputs relevant information and proceeds to the image acquisition interface to capture the sample image, as depicted in Figure 6A. The green box represents the viewfinder, where the custom app analyzes and calculates the color values within this area. To minimize errors introduced by human modifications, the first five sample images captured are standard products used to establish the color-concentration equation. The concentration of the sample to be measured is then calculated using this equation, as illustrated in Figure 6B.

In regions with limited medical resources, standard laboratory equipment may not be readily available. To address this issue, the app dynamically adjusts the size of the viewfinder frame to accommodate various experimental conditions, as shown in Figure 6C. This flexibility allows for the adaptation of different experimental devices, including slit cuvettes, centrifuge tubes, and even syringe-needle protective sleeves. Experimental results obtained using these diverse utensils have proven to be satisfactory.

### 3.3. Sample Testing

(i) Comparison of smartphone test results.

To validate the proposed method, gradient concentrations of AFP were added to the prepared functionalized AuNPs, as detailed in Table 1. AFP concentrations were configured in the range of 50–200 ng/mL in increments of 50 ng/mL. The left column represents the prepared standards, while the right column shows the corresponding results obtained from the app test. Overall, the colorimetric detection method yielded results within an acceptable range. However, a relatively large error occurred in the test results when the standard concentration was 150 ng/mL. This discrepancy was attributed to vibrations generated by the hardware platform during testing, causing the position of the sample to shift. As a result, part of the white hardware material was captured by the viewfinder, and its gray value (white) was close to 255. This led the app to overestimate the actual AFP concentration value during calculation.

Two improvement measures can be implemented to address this issue. Firstly, the hardware platform could be enhanced by incorporating a slot to securely hold the sample in place. This would minimize the shift of the sample caused by vibrations. Additionally, new image-recognition algorithms could be developed to automatically identify sample images. In actual experimental images, the gray value of the background color (white) was significantly higher than the range of gray values for the sample (70–130). Therefore, during the calculation process, a threshold could be applied to determine whether a pixel belongs to the sample image or the background image. For instance, pixels with a gray value less than 150 could be considered part of the sample image, while those with higher gray values would be categorized as background pixels and not included in the calculation. This improved method would further enhance the accuracy of the algorithm during the calculation process.

(ii) Limit of detection (LOD).

The detection accuracy and the limit of detection are important parameters for measuring colorimetric sensing based on a smartphone. To obtain the minimum fluctuation deviation, in the absence of detection samples, the spectrum of non-functionalized gold colloidal nanoparticles was tested, and it was found that the resonance wavelength fluctuation was only 0.025 nm under the interference of PBS. The limit of detection can be obtained by fitting the slope of the curve together with Figure 6B.

According to the definition of IUPAC [32], the SNR (signal-to-noise ratio) method is used in this study to calculate the detection limit, which is calculated by dividing the slope by three times the standard deviation of multiple blank solutions. The formula is shown as follows:

(7) LOD=3σ/s

where σ is the standard deviation and *s* is the slope of the fitted working curve.

When σ=0.025 nm and *s* = 0.9, the LOD=0.083 ng/mL. 

The detection process for the AFP antigen varies depending on the method used, with differences in detection limits and linear ranges, as shown in Table 2. In this experiment, a smartphone-based colorimetric detection method was employed, which has a much lower detection limit (0.083 ng/mL) compared to the traditional ELISA method (2 ng/mL). Additionally, the detection process is relatively simpler, and the cost is significantly lower.

(iii) Accuracy.

According to Table 1, it can be calculated that the average detection accuracy of smartphone-based colorimetric sensing can reach 90.81%. In practice, the detection accuracy of smartphone-based specific sensor components and systems is related to many factors. This is mainly reflected in the following points:

A. Sensor performance: The sensitivity and response speed of the sensor are important factors affecting the detection accuracy. High-performance sensors enable the more accurate detection of small color changes.

B. Detection instrument: The accuracy and camera resolution of the detection instrument (smartphone) used to read the color change will directly affect the accuracy of the detection results.

C. Sample preparation: The preparation process, volume, and uniformity of the sample will affect the accuracy of the test. The purity and consistency of the sample are also key factors.

D. Environmental conditions: Environmental light, temperature, humidity, and other factors will affect the performance of the colorimetric sensor, thus affecting the detection accuracy. When used, it is necessary to measure under stable environmental conditions.

E. Data processing: Data processing methods (such as correction, standardization, etc.) are also very important for improving detection accuracy. Accurate data processing can effectively reduce noise and error.

F. Repeatability.

For biochemical sensors, repeatability is an important index. In colorimetric sensing based on gold nanoparticles, the repeatability can reach more than 90% within 2 weeks of reagent preparation, according to the limitation of reaction time. When the gold nanostructure is functionalized and binds to the antibody for too long (more than 4 h), the gold–sulfur bond on the surface of the gold nanostructure will dissociate, which will eventually lead to the failure of the experiment, and the repeatability will become poor. Factors that affect the repeatability of test results are as follows:

A. Preparation of gold nanoparticles: Considering the consistency of particle size and shape, the size and shape of gold nanoparticles have a significant effect on their optical properties. Therefore, the size and shape of the particles need to be strictly controlled during the preparation process to ensure the consistency of the sensing results.

B. Surface modification and functionalization: The modifiers and functionalized molecules on the surface of gold nanoparticles have a great influence on their colorimetric response. Variations in the surface modification process may lead to differences in repeatability.

C. pH value of the solution: The pH value of the solution can affect the dispersion and stability of the gold nanoparticles, thus affecting the results of colorimetric sensing.

D. Sample preparation process: The sample preparation process needs to be strictly standardized to reduce human error and variation.

E. Calibration of optical testing instruments: The optical testing instruments used need to be calibrated regularly to ensure the accuracy and consistency of the test results.

F. Light source stability: The instability of the light source may lead to fluctuations in the detection signal, thus affecting repeatability.

G. Consistency of data processing methods: Data processing and analysis methods need to be standardized to ensure comparability between different experimental results.

H. Signal-to-noise ratio: A higher signal-to-noise ratio can improve the reliability and repeatability of the test results.

By strictly controlling the above factors, the repeatability and reliability of colorimetric sensing based on gold nanoparticles can be improved. Developing and following standardized operating procedures (Sops) is key to ensuring repeatability during experimental design and operation.

## 4. Conclusions 

Using smartphones for the quantitative detection of target analytes is an economical and efficient approach. In this study, smartphones were employed for the quantitative detection of target analytes, particularly by using colorimetry to analyze color changes resulting from variations in analyte concentration. A custom hardware platform and mobile application were developed to work in tandem, detecting the color change of functionalized gold nanoparticles (AuNPs) after the addition of alpha-fetoprotein (AFP). The hardware platform provided uniform illumination, thereby enhancing the accuracy of the detection process. The mobile application included a dynamically adjustable viewfinder, allowing it to adapt to various experimental setups. Compared to traditional ELISA methods, this approach achieved a detection limit as low as 0.083 ng/mL, which is lower than the traditional ELISA detection limit.

This smartphone-based colorimetric sensing method offers several advantages: (1) low cost—the method is economical, making it accessible for widespread use; (2) high efficiency—the process is designed to be fast, reducing the time required for analysis; (3) user-friendly—the method is easy to use, which is crucial for application in various environments; and (4) strong portability—the system is compact and easy to carry, making it highly suitable for use in remote or resource-limited areas.

Despite these advantages, there is still room for further improvement. Future enhancements may include reducing the size of the hardware platform and improving accuracy. The design of a new hardware platform has already begun, with the size reduced by approximately half through modifications to the light source. As previously mentioned, improving the accuracy of the algorithm by implementing a threshold mechanism remains a key focus for further optimization.

## Figures and Tables

**Table 1:** Comparison of smartphone test results with actual calibration concentrations.

AFP Concentration (ng/mL)	Testing Result (ng/mL)	Deviation Value (ng/mL)	Accuracy
50	56.2	6.2	87.6%
100	107.2	7.2	92.8%
150	162.3	12.3	91.8%
200	217.9	17.9	91.05%

**Table 2:** Comparison of AFP detection ability of different methods.

Method	Detection Element	Linear Range	LOD	Sample	Ref
Paper-based colorimetry	Antibody	0.1–100 ng/mL	1.054 ng/mL	Serum	[33]
Two-site ELISA #	Affibody	6–100 ng/mL	2 ng/mL	Serum	[34]
LSPR combined immunoassay	Antibody	20–200 ng/mL	24 ng/mL	Serum	[28]
Fluorescence immunoassay	Antibody	1.0–4.0 ng/mL	41 pg/mL	Serum	[35]
Photoelectrochemical immunoassay	Antibody	0.01–50 ng/mL	1.2 pg/mL	Serum	[36]
Colorimetric ELISA	Antibody	15–600 ng/mL	0.82 ng/mL	Serum	[37]
Smartphone colorimetry	Antigen	10–300 ng/mL	0.083 ng/mL	Serum	This work

#ELISA: Enzyme-linked immunosorbent assay.

## Data Availability

The original contributions presented in the study are included in the article, further inquiries can be directed to the corresponding author.
